# Evaluation of predictive performance of fetal urinary inflammatory markers of postnatal kidney function in fetuses with posterior urethral valves

**DOI:** 10.1007/s00467-024-06608-x

**Published:** 2024-11-30

**Authors:** Nicolas Geraud, Audrey Casemayou, Melinda Alves, Benjamin Breuil, Marcin Tkaczyk, Małgorzata Stańczyk, Krzysztof Szaflik, Tomasz Talar, Stéphane Decramer, Julie Klein, Joost P. Schanstra, Bénédicte Buffin Meyer

**Affiliations:** 1 National Institute of Health and Medical Research (INSERM), UMR 1297, Institute of Metabolic and Cardiovascular Disease, Toulouse, France; 2 University Paul Sabatier, Toulouse III, Toulouse, France; 3 Centre De Référence Des Maladies Rénales Rares du Sud-Ouest (SORARE), Toulouse University Hospital, Toulouse, France; 4 Department of Pediatric, Immunology and Nephrology, Polish Mother’s Memorial Hospital Research Institute, Łódź, Poland; 5 Department of Gynecology, Fertility and Fetal Therapy, Polish Mother’s Memorial Hospital Research Institute, Łódź, Poland; 6 Department of Neonatology and Intensive Therapy, Polish Mother’s Memorial Hospital Research Institute, Łódź, Poland; 7 Department of Pediatric Internal Medicine, Rheumatology and Nephrology, Toulouse University Hospital, Toulouse, France

**Keywords:** Prenatal diagnosis, Biomarker, Body fluids, Outcome, Kidney function, Inflammation

## Abstract

**Background:**

There are proposed roles for inflammation in the development of congenital obstructive uropathy in the setting of posterior urethral valves (PUV). However, the value of inflammatory proteins as predictive markers of postnatal kidney function, key in the management of fetuses with PUV, has not been explored. We screened fetal urine of fetuses with PUV with a panel of inflammatory proteins to determine their predictive value of postnatal kidney function.

**Methods:**

Twenty-five different chemokines and cytokines were measured using a multiplex immunoassay in fetal urine of 79 PUV patients from retrospective cohorts, separated in discovery (*n* = 52) and validation (*n* = 27). The candidate markers were also quantified in amniotic fluid samples obtained from 16 PUV and 25 other congenital anomalies of the kidney and the urinary tract pregnancies. The performance of validated candidate inflammatory proteins was compared to the previously published 12PUV fetal urine peptide signature.

**Results:**

Fetal urine chemokines CCL2 (MCP-1), CXCL9 (MIG), and CCL4 (MIP-1β) were identified as predictive of postnatal kidney failure in fetuses with PUV from the discovery cohort. Their predictive potential was confirmed in the validation cohort (AUCs of 0.87, 0.81, and 0.86, respectively). The performance of these individual chemokines was lower than the previously published 12PUV fetal urine peptide signature. However, the combination of the three chemokines performed similarly to 12PUV. In contrast, these three chemokines were not predictive of outcome in amniotic fluid.

**Conclusions:**

We identified chemokines in fetal urine of PUV pregnancies that, after external validation, could serve as predictive biomarkers of postnatal outcome and contribute to improve prenatal PUV management.

**Graphical Abstract:**

**Figure:**
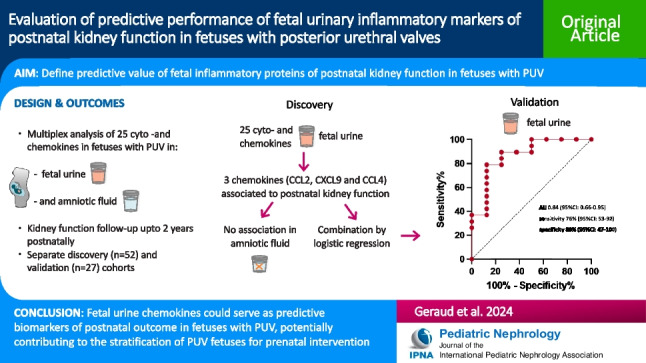
A higher resolution version of the Graphical abstract is available as Supplementary information

**Supplementary Information:**

The online version contains supplementary material available at 10.1007/s00467-024-06608-x.

## Introduction

Posterior urethral valves (PUV) are associated with a diverse spectrum of outcomes including severe kidney phenotypes leading to prenatal death, but also live-born children with normal kidney function. Although uncommon, PUV accounts for 6 to 29% of pediatric patients with kidney failure (KF) [1, 2].

Currently, PUV is one of the rare congenital anomalies of the kidney and the urinary tract (CAKUT) where prenatal intervention is possible. This intervention focuses on decompression of the bladder by either vesico-amniotic shunt (VAS) or fetal endoscopic valve ablation. However, while systematic reviews indicate that VAS increases perinatal survival, its effect on 1–2-year survival and outcome of kidney function remains uncertain [3–5]. Consequently, research has concentrated on methods of selecting fetuses with PUV whose postnatal kidney function is likely to be normal or mildly impaired and for whom prenatal intervention could be beneficial to avoid further deterioration of fetal kidney function and the onset of pulmonary hypoplasia. To identify such fetuses, Ruano et al. [6] have proposed to use a score based on ultrasound and fetal urine electrolytes. Nevertheless, the aforementioned score remains to be validated, particularly in the light of several meta-analyses showing the absence of predictive value of ultrasound and fetal urine biochemistry used for over several decades [7–9]. Klein et al. identified a fetal urinary signature composed of 12 peptides (12PUV) using mass spectrometry, which demonstrated excellent predictive performance for the postnatal onset of KF at 2 years of age [10]. This signature demonstrated superior performance compared to conventional parameters including ultrasound-measured characteristics of the fetal kidneys (e.g., echogenicity, cortico medullary differentiation, kidney size) and amniotic fluid volume as well as fetal urine biochemistry (sodium, β2-microglobulin). This signature was successfully validated in an independent single center study involving 23 PUV pregnancies from Poland [11]. It is also currently undergoing multicenter international validation involving > 30 different European centers [12]. Despite the promise of the 12PUV fetal urinary peptide signature, its routine deployment may be hindered by the high technicality of its analysis, which involves mass-spectrometry analysis that is not accessible to all clinics. It is therefore interesting to study in parallel other options.

The available experimental evidence indicates that inflammation plays an important role in the development of obstructive uropathies (OU) [13]. In addition, studies have demonstrated that the levels of inflammatory proteins in fetal urine from fetuses with PUV are significantly higher than in matched fetal urine from healthy preterm neonates [14, 15]. However, although clearly connected to the pathophysiology of OU, the predictive value ability with respect to postnatal kidney outcome of inflammatory proteins in fetal body fluids has never been evaluated. This could contribute to the management of pregnancies involving fetuses with PUV. Here we examined a panel of 25 inflammatory proteins, composed of chemokines and cytokines, using a multiplex immunoassay in prenatal body fluids from 79 fetuses with PUV and analyzed their predictive potential with respect to postnatal kidney outcome.

## Materials and methods

### Patients

**Fig. 1:**
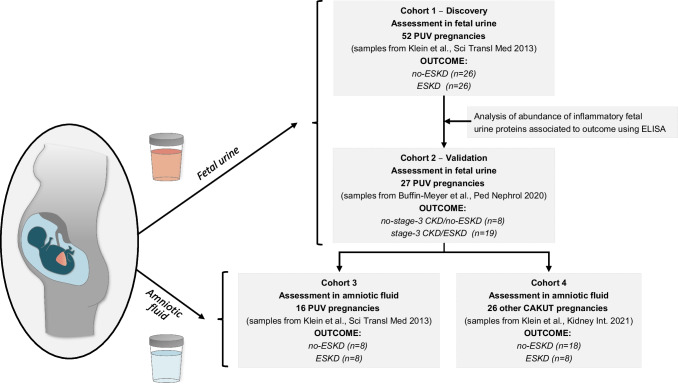
Study setup. Our study was based on archival samples of three independent retrospective studies involving patients with PUV. Abbreviations: KF, kidney failure before the age of 2 years; no-KF, absence of kidney failure before the age of 2 years; CKD stage 3/KF, stage 3 chronic kidney disease or KF before the age of 6 months; CKD stage 3/no-KF, absence of stage 3 chronic kidney disease or kidney failure before the age of 6 months

This is a retrospective case–control study using fetal urine and amniotic fluid samples stored at − 80 °C of previously published studies in 2013, 2020, and 2021 [10, 11, 16] (Fig. 1). Samples were obtained in multiple French and one Polish centers.

Fetal urine or amniotic fluid was collected from fetuses with confirmed PUV at birth between gestational ages of 14.0- and 35.5-week amenorrhea (wa) and 14.6–37 wa, respectively. Amniotic fluid was also collected from fetuses with CAKUT between 16 and 39 wa. The main outcome was development of KF at 2 years or chronic kidney disease stage 3 (CKD, eGFR < 60 mL/min/1.73, Schwartz formula) at 6 months during post-natal follow-up. Fifty-three percent and 31% of the fetuses with PUV or CAKUT developed CKD stage 3 or KF, respectively. Postnatal follow-up was 6 to 24 months depending on the study. The study size was based on the availability of samples for this rare disease. IRB approvals: cohorts 1 and 3 were historical samples obtained before 2010 and according to French law at that time were obtained with a consent only; cohort 2: Lodz, Poland No. 1/2016; cohort 4: Toulouse, France NO. RCB 2010-AO1151-38.

### Multiplex immunoassay

The Legendplex (Biolegend) human proinflammatory chemokine panel 1 (CXCL8 (IL-8), CXCL10 (IP-10), CCL11 (Eotaxin), CCL17 (TARC), CCL2 (MCP-1), CCL5 (RANTES), CCL3 (MIP-1α), CXCL9 (MIG), CXCL5 (ENA-78), CCL20 (MIP-3α), CXCL1 (GROα), CXCL11 (I-TAC), CCL4 (MIP-1β)), and human inflammation panel 1 (IL-1β, IFN-α2, IFN-γ, TNF-α (TNFSF2), CCL2 (MCP-1), IL-6, CXCL8 (IL-8), IL-10, IL-12p70, IL-17A, IL-18, IL-23, IL-33) were used according to the manufacturer’s instructions. Samples were diluted twofold in the provided assay buffer. Flow cytometry analysis was performed on a BD Fortessa LSRII.

### Statistics

Statistical analysis was performed using GraphPad Prism (V10.0.2 (171)) or Medcalc (Version 20, 64 bit). Patient characteristics were compared with respect to the data of the discovery cohort using a Fisher’s exact or a Chi-squared test. Two group comparisons were assessed the Mann–Whitney test. Predictive performances were assessed by calculating the area under the receiver operating characteristic curve (AUC) and 95% confidence interval (DeLong method). Sensitivities and specificities were calculated taking the threshold identified by the Youden index in the discovery cohort. These thresholds were then used to calculate the sensitivities and specificities in the validation cohort. These sensitivities and specificities were compared using the McNemar test. Differences between groups were analyzed by a one-way ANOVA analysis with a Tukey post hoc test with a *p* < 0.05 considered significant. Pearson’s correlation analysis was performed to analyze correlation between gestational age and chemokine abundance with a *p* < 0.05 considered significant.

## Results

### Patients and study setup

Four distinct retrospective cohorts were utilized for the study (Fig. 1). The initial analysis of the abundance of the fetal urinary inflammatory proteins in fetuses with PUV involved 52 patients developing or not KF at 2 years of age (discovery, cohort 1, patients from the Klein et al., study [10]). The differential abundance of inflammatory fetal urine proteins with respect to postnatal kidney function was validated in the second cohort of 27 PUV pregnancies (validation, cohort 2, patients from the Buffin-Meyer et al., study [11]). In this cohort [11], the postnatal follow-up period was 6 months. Accordingly, the endpoint was modified to at least stage 3 CKD (eGFR < 60 mL/min/1.73 m^2^, Schwartz formula) or perinatal death. This was done on the basis of the hypothesis that CKD stage 3 at the age of 6 months is a predisposition to progression to severe KF at 2 years of age. In the last step, the abundance of the inflammatory proteins associated with kidney outcome (KF or no-KF at the age of 2 years) was evaluated in amniotic fluid obtained from16 PUV and 26 other CAKUT pregnancies (cohort 3, patients from the Klein et al., 2013 study [10] and cohort 4, patients from the Klein et al., 2021 study [16], respectively).

A detailed account of the patient characteristics can be found in Table 1. Overall, there were no major differences between the cohorts except that the gestational age (GA) upon discovery of PUV was lower in the validation cohort (GA of 27.2 [14.6–35.20] versus 22.1 [13.0–35.5] weeks amenorrhea (wa), *p* = 0.0005, Table 1) and ultrasound (US) abnormalities were different (*p* = 0.001, Table 1). With respect to US-abnormalities, the discovery cohort exhibited a higher prevalence of patients with normal kidneys, with cystic kidneys and hypoplasia, compared to the validation cohort and a lower proportion of patients with hyperechogenic kidneys.

**Table 1:** Clinical characteristics of patient populations

	Fetal urine			Amniotic fluid			
	PUV, discovery (*N* = 52) Cohort 1	PUV, validation (*N* = 27) Cohort 2	*P* value^‡^	PUV (*N* = 16) Cohort 3	*P* value^‡^	Other CAKUT(*N* = 26) Cohort 4	*P* value^‡^
**Gestational age at discovery of kidney abnormalities,** *mean [range]*	27.2 [14.6–35.2]	22.1 [13.0–35.5]	0.0005^$^	27.5 [14.6–37.0]	0.898^$^	26.3 [16.0–39.0]	0.463^$^
**Outcome**			0.098^£^		> 0.999^£^		0.147^£^
* no-CKD stage 3/no-KF, number*	26	8		8		18	
* CKD stage 3/KF, number*	26	19		8		8	
**Ultrasound: amniotic volume**			0.784^&^		€		€
* Normal, number*	28	14		9		14	
* Oligohydramnios, number*	12	8		4		4	
* Anhydramnios, number*	12	5		2		4	
* Not available, number*	0	0		3		4	
**Ultrasound: kidney abnormalities**			0.001^&^		€		€
* Normal, number*	20	6		7		0	
* Hyperechogenicity, number*	7	16		2		12	
* Cysts, number*	17	4		0		1	
* Hypoplasia, number*	6	2		5		4	
* Not available, number*	2	0		4		0	
* Other, number*	0	0		0		9	

^‡^Compared to cohort 1; ^$^*t* test; ^£^Fisher’s exact test; ^&^chi-squared test (rows with a 0 value are not taken into account Fisher’s exact and chi-squared test); ^€^not enough data to perform analysis

### Fetal urinary CCL2, CXCL9, and CCL4 are predictive of postnatal kidney outcome in fetuses with PUV

**Fig. 2:**
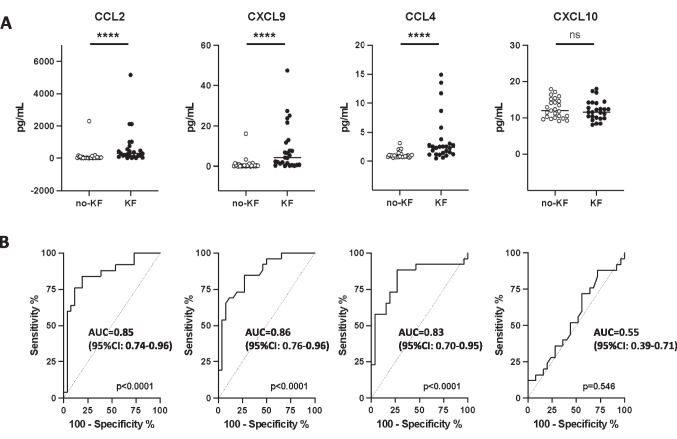
Abundance of chemokine CCL2, CXCL9, CCL4, and CXCL10 in fetal urine of patients with PUV in the discovery cohort (cohort 1 in Fig. 1). **A** CCL2, CXCL9, and CCL4 are significantly more abundant in fetal urine of PUV patients developing post-natal KF. CXCL10, randomly chosen as a negative control among the 22 inflammatory proteins, displays a similar abundance in both outcomes. **B** Receiver operator curves (ROC) for the four fetal urine chemokines. Abbreviations: ****p < 0.0001; ns, non-significant; KF, kidney failure before the age of 2 years (*n* = 26); no-KF, absence of kidney failure before the age of 2 years (*n* = 26); AUC, area under the curve

The abundance of 22 fetal urinary inflammatory proteins (CCL5, CXCL8, and IL-18 were not detectable) and their association with postnatal kidney function was measured from 52 fetuses with PUV in the discovery cohort (cohort 1 in Fig. 1, Table 1, [10]). The fetal urinary abundance of CXCL8, CXCL10, CCL11, CCL17, CCL5, CCL3, CXCL5, CCL20, CXCL1, CXCL11, IL-1β, IFN-α2, IFN-γ, TNF-α, IL-6, CXCL8, IL-10, IL-12p70, IL-17A, IL-18, IL-23, and IL-33 was not modified by kidney outcome (Supplementary Fig. S1 and Fig. 1 for CXCL10). In contrast, CCL2, CXCL9, and CCL4 were significantly more abundant in fetal urine of patients with postnatal KF (Fig. 2a, *P* < 0.0001) than in urine of fetuses without postnatal KF. The AUC of associated ROC curves were 0.85 (95%CI: 0.74 to 0.96), 0.86 (95%CI: 0.76 to 0.96), and 0.82 (95%CI: 0.70 to 0.95), respectively (Fig. 2b). The selection of a cut-off according to the Youden index yielded a sensitivity of 84% (95%CI: 65–96) and specificity of 81% (95%CI: 61–93) for CCL2, a sensitivity of 65% (95%CI: 44–83) and specificity of 92% (95%CI: 75–99) for CXCL9 and a sensitivity of 89% (95%CI: 69–97) and specificity of 73% (95%CI: 52–88) for CCL4. CCL2 and CXCL9 remained significant after correction for multiple testing (Benjamini–Hochberg corrected *p* value < 0.05) and also after normalization for the total fetal urine protein content (Supplementary Fig. S2).

**Fig. 3:**
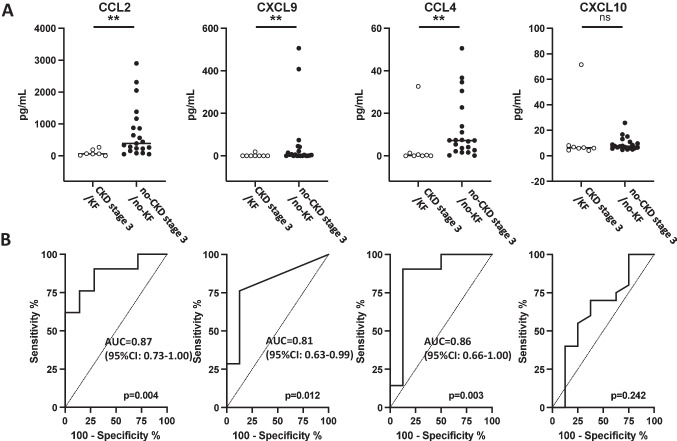
Abundance of chemokine CCL2, CXCL9, CCL4, and CXCL10 in fetal urine of patients with PUV in the validation cohort (cohort 2 in Fig. 1).** A** CCL2, CXCL9, and CCL4 are significantly more abundant in fetal urine of PUV patients developing post-natal CKD stage 3/KF. CXCL10, randomly chosen as a negative control among the 22 inflammatory proteins, displays a similar abundance in both outcomes. **B** Receiver operator curves (ROC) for the four fetal urine chemokines. Abbreviations: **p < 0.01; ns, non-significant; CKD stage 3/KF, stage 3 chronic kidney disease or kidney failure before the age of 6 months (*n* = 19); no-CKD stage 3/no-KF, absence of stage 3 chronic kidney disease or kidney failure before the age of 6 months (*n* = 8); AUC, area under the curve

In the next step, we aimed to validate these observations in an independent, albeit smaller, cohort. To this end, the concentration of CCL2, CXCL9, and CCL4 was measured in fetal urine samples from 27 fetuses with PUV (validation cohort 2 in Fig. 1, Table 1 [11]). The increased abundance of the three chemokines in fetal urine of fetuses with PUV developing CKD stage 3/KF before 6 months of age (compared to fetuses without postnatal CKD stage 3/KF) was confirmed (Fig. 3). The associated predictive potential of the three molecules was also confirmed in this validation cohort. Indeed, CCL2 displayed an AUC of 0.87 (95%CI: 0.73–1.00) and, based on the cutoff previously defined in the discovery cohort (Supplementary Fig. S3), a sensitivity of 91% (95%CI: 70–99) and a specificity of 63% (95%CI: 25–92). CXCL9 displayed an AUC of 0.81 (95%CI: 0.63–0.99), a sensitivity of 55% (95%CI: 32–77) and a specificity of 88% (95%CI: 47–100). CCL4 exhibited an AUC of 0.86 (95%CI: 0.66–1.00), a sensitivity of 90% (95%CI: 70–99) and a specificity of 75% (95%CI: 35–97). CXCL10, a randomly chosen other inflammatory protein that was not associated with postnatal kidney function outcome in the discovery cohort (Fig. 2a, b), was still not predictive of outcome in fetal urine of the patients in the validation cohort thereby serving as negative control (Fig. 3).

Additionally, the link between the abundance of the three chemokines and gestational age or ultrasound parameters was examined in both the discovery and validation cohorts. The three chemokines failed to correlate with gestational age (Pearson’s *r* = 0.11, *p* = 0.34 for CCL2; *r* = − 0.04, *p* = 0.75 for CxCL9; and *r* = − 0.23, *p* = 0.10 for CCL4). Moreover, with the exception of an increased abundance of CCL2 in patients with anhydramnios (*P* = 0.03), no significant association was observed between the abundance of the three chemokines and the ultrasound kidney parameters, including hyperechogenicity, presence of cysts, or hypoplasia (Supplementary Fig. S4).

The three chemokines were significantly correlated with each other (CCL2 versus CXCL9, Pearson correlation *r* = 0.70 (*p* = 1.1e-008); CCL2 versus CCL4, *r* = 0.45 (*p* = 0.0007); and CCL4 versus CXCL9, *r* = 0.54 (*p* = 0.00005). However, as these correlations were not perfect, we hypothesized that a combination of the chemokines might further improve the prediction of postnatal kidney outcome. Combination of the fetal urinary abundance of CCL2, CXCL9, and CCL4 using logistic regression (logit (*p*) = − 2.49805 + 1.12575 × CXCL9 + 0.7585 × CCL4 + − 0.0013076 × CCL2) led to an AUC of 0.88 (95%CI: 0.76–95), a sensitivity of 69% (95%CI: 48–86) and a specificity of 100% (95%CI: 86–100) in the discovery cohort (cohort 1, Fig. 1). In the validation cohort (cohort 2 in Fig. 1), the combination led to an AUC of 0.84 (95%CI: 0.66–0.95), a sensitivity of 76% (95%CI: 53–92) and a specificity of 88% (95%CI: 47–100). However, these performances were no better than those of the individual molecules (Supplementary Table S1).

The collective data indicate that fetal urine CCL2, CXCL9, and CCL4 may serve as potential biomarkers for postnatal kidney function prediction in fetuses with PUV.

### The amniotic fluid abundance of CCL2, CXCL9, and CCL4 is not associated with postnatal kidney outcome in fetuses with PUV

**Fig. 4:**
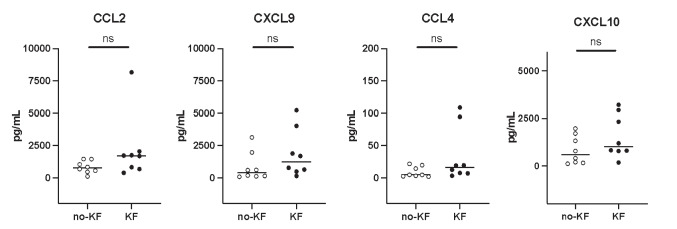
Abundance of selected chemokines in amniotic fluid of PUV patients (cohort 3 in Fig. 1). The abundance in amniotic fluid of CCL2, CXCL9, and CCL4 and the fetal urine negative control inflammatory protein CXCL10 was not different in fetuses with early KF versus fetuses not developing postnatal KF. Abbreviations: ns, non-significant. KF, kidney failure before the age of 2 years (*n* = 8); no-KF, absence of kidney failure before the age of 2 years (*n* = 8)

It has recently been demonstrated that amniotic fluid shares 55–65% of its content in small proteins with fetal urine [17]. Accordingly, in order to potentially reduce the invasive character of a test based on fetal urine, we also studied the abundance of the three selected candidate chemokines in amniotic fluid of 16 fetuses from the cohort 3 (Fig. 1). The abundance of CCL2, CXCL9, and CCL4, as well as the fetal urine negative control inflammatory protein CXCL10, was not different in amniotic fluid of fetuses (*n* = 8) with postnatal KF compared to fetuses (*n* = 8) not developing KF before 2 years of age (Fig. 4). These four inflammatory amniotic fluid proteins were also not associated with postnatal kidney outcome in fetuses having CAKUT other than PUV (cohort 4 in Fig. 1, Supplementary Fig. S5).

### Comparison with the 12PUV fetal urinary peptide signature

Subsequently, we compared CCL2, CXCL9, and CCL4 as well as their combination with the previously published [10] and validated [11] 12PUV fetal urinary peptide signature (cohort 2 in Fig. 1). The AUCs of the three chemokines or their combination were not significantly different from that of the 12PUV signature (Supplementary Table S1). CCL2 and CCL4 displayed similar sensitivity than 12PUV to predict outcome but had a significantly lower specificity (88% (95%CI: 47–100) for 12PUV versus 63% (95%CI: 25–92), *p* < 0.01 and 75% (95%CI: 35–97), *p* < 0.05, respectively) (Table 2). In addition, CXCL9 exhibited a markedly lower sensitivity than 12PUV (89% (95%CI: 70–99) for 12PUV versus a sensitivity of 55% (95%CI: 32–77), *p* < 0.05 for CXCL9), but with a similar specificity (Table 2).

**Table 2:** Validation of sensitivity and specificity of inflammatory markers in the validation cohort (cohort 2) [11] and comparison to the 12PUV classifier [10]

	Sensitivity	Specificity
CCL2	91% (95%CI: 70–99)	63% (95%CI: 25–92)**
CXCL9	55% (95%CI: 32–77)*	88% (95%CI: 47–100)
CCL4	90% (95%CI: 70–99)	75% (95%CI: 35–97)*
Combo CCL2/CXCL9/CCL4	76% (95%CI: 53–92)	88% (95%CI: 47–100)
12PUV	89% (95%CI: 70–99)	88% (95%CI: 47–100)

*P < 0.05; **P < 0.01 compared to 12PUV using the McNemar test for comparison of sensitivities and specificities

However, the sensitivity and specificity of the combination of CCL2/CXCL9/CCL4 versus 12PUV were not found to be significantly different (Table 2).

## Discussion

Early experimental studies have pointed to a major inflammatory response in OU [13]. These findings have been confirmed in fetuses with PUV by the accumulation of a number of chemokines and cytokines in fetal urine [14, 15]. Furthermore, the accumulation of inflammatory and profibrotic cytokines in urine has also been documented in other forms of OU, including ureteropelvic junction obstruction [18, 19] and vesicoureteral reflux [20]. However, in all studies, patients with OU were compared to healthy controls. Although this clearly confirms a major inflammatory response to OU, it does not link this inflammatory response to kidney outcome. In the present study, we observed among a panel of 25 tested fetal urinary chemokines and chemokines that a combination of fetal urine chemokines CCL2 (MCP-1), CXCL9 (MIG), and CCL4 (MIP-1β) was strongly predictive of the development of CKD stage 3/KF after birth in fetuses with PUV. Following external validation, the application of this chemokine signature, alone or in combination with stratification strategies based on clinical data [6], would enable the stratification of fetuses whose postnatal kidney function will be most likely normal or mildly impaired for VAS or fetal endoscopic valve ablation to avoid further degradation of fetal kidney function. To reduce the potentially invasive nature of the analysis, we also examined the association of these three chemokines with outcome in amniotic fluid in PUV pregnancies. However, the three chemokines failed to show association with postnatal kidney outcome in amniotic fluid. This might be due to the fact that fetal urine is not the only source of amniotic fluid and/or that the number of patients with available amniotic fluid was too low (*n* = 8 in each group).

CCL2 plays a key role in the regulation of monocytes in inflammatory diseases by guiding monocytes into inflamed tissue via a chemokine gradient [21]. It has been shown to play an important role in the progression of CKD in animal models [22] and urinary CCL2 has been associated with progression of CKD in both adults and children [23, 24]. We show here that this appears to be similar in a prenatal context during kidney development, thereby generalizing the role of CCL2 in kidney disease and expanding it to kidney development. In contrast, the association of the CXCL9 and CCL4 with kidney development/progressive kidney disease is novel. The chemokine network is extremely complex [21] and CCL2, CCL4, and CXCL9 act on different chemokine receptors. Therefore, a specific involvement of each chemokine and associated receptors in PUV cannot be determined at this time.

Although CCL2, CCL4, and CXCL are predictive of postnatal kidney outcome, they do not perform better, when taken individually, than the previously mass spectrometry defined fetal urine peptide signature composed of 12 peptides [10, 11]. However, the logistic regression analysis demonstrated that the combination of the three chemokines yielded comparable results to 12PUV. This finding requires confirmation in a larger cohort. Nevertheless, if the predictive efficacy of the combination of the three chemokines is confirmed on a larger scale, implementing an immunoassay for the three chemokines may be more straightforward than implementing the mass spectrometry–based 12PUV signature. Indeed, immunoassay-based assays are readily available in clinical laboratories (even in a multiplex configuration) and require less expertise than mass spectrometry–based strategies.

It is noteworthy that despite the disparate fetal kidney ultrasound characteristics observed between the discovery and validation cohorts (Table 1), this did not influence the predictive capacity of fetal urinary chemokines for postnatal kidney outcomes. Moreover, no enrichment of chemokines was observed in fetuses with specific fetal kidney abnormalities, such as cysts or hypodysplasia. This finding is consistent with the observation of urinary peptides in amniotic fluid of patients with CAKUT, which are also predominantly associated with postnatal kidney outcome, but not with the CAKUT disease etiology based on ultrasound. [16]. These observations also corroborate data obtained in a meta-analysis demonstrating the absence of a clear predictive value of ultrasound for postnatal kidney function outcome in congenital lower urinary tract obstruction [25].

The peptides in the 12PUV signature consist of the major part of collagen fragments (11 out of 12) that was hypothesized to be associated with connective tissue remodeling in the PUV kidneys. On the other hand, peptide fragments of chemokines were not identified as markers of postnatal kidney outcome in PUV pregnancies [10]. This is probably due to the fact that the chemokine peptides are not detected by mass spectrometry due to the higher detection threshold of mass spectrometry in the ng/mL range [26], versus lower pg/mL range in antibody-based strategies [27, 28].

In addition to the aforementioned collagen fragments and chemokines as predictive markers of postnatal kidney outcome, additional candidates could be evaluated. In that context, Bastos et al. [14] observed modified abundance of typical glomerular and tubular markers including epidermal growth factor (EGF), calbindin, osteoactivin, kidney injury molecule 1 (KIM-1), factor of trefoil 3, cystatin C, and renin in fetal urine of patients of PUV compared to healthy age-matched controls. It would be interesting to evaluate the performance of such molecules in a predictive setting. For example, decreased EGF has already been identified as a potential biomarker of CKD progression [29, 30].

While our study offers insight into the potential of chemokines as predictive biomarkers of postnatal kidney outcome in PUV, it has limitations. The study was retrospective, and validation in a prospective cohort is desirable. In addition, our retrospective validation cohort (cohort 2 in Fig. 1) was relatively small especially with respect to patients with a favorable outcome (*n* = 8). Another limitation is the difference in postnatal follow-up in the discovery (2 years) and validation (6 months) cohort. These three limitations could be removed by external validation in the ongoing prospective ANTENATAL study that aims to include 300 European PUV pregnancies and assess the potential predictive value of body fluid markers for postnatal kidney outcome [12].

In conclusion, we identified chemokines in fetal urine of PUV pregnancies that could serve as predictive biomarkers of the postnatal outcome and improve the prenatal management of PUV.

## Supplementary Information

Below is the link to the electronic supplementary material.

Supplementary file1 (DOCX 777 KB)

Graphical abstract (PPTX 146 KB)

## Data Availability

The data sets generated during and/or analyzed during the current study are available from the corresponding authors on reasonable request. It will contain deidentified participant data.
