# Supplementary material for: Evaluation of predictive performance of fetal urinary inflammatory markers of postnatal kidney function in fetuses with posterior urethral valves
Source: Pediatr Nephrol. 2024 Nov 30;40(4):1023–32. doi: 10.1007/s00467-024-06608-x (PMC11885327; doi:10.1007/s00467-024-06608-x)
Supplement: Supplementary file 1 — Supplementary file1 (DOCX 777 KB) [file 467_2024_6608_MOESM1_ESM.docx]

**Supplement to:**

**Evaluation of predictive value of fetal urinary inflammatory markers of**

**postnatal kidney function in fetuses with posterior urethral valves**

*Nicolas Geraud^1,2^, PhD, Audrey Casemayou^1,2,3^ PhD, Melinda Alves^1,2^, Benjamin Breuil^1,2^, Marcin Tkaczyk^4^ MD PhD, Małgorzata Stańczyk^4^ PhD, Krzysztof Szaflik^5^ MD PhD, Tomasz Talar^6^ MD PhD, Stéphane Decramer^1,2,3,7^ MD PhD, Julie Klein^1,2^ PhD, Joost P Schanstra^1,2*^ PhD, Bénédicte Buffin Meyer^1,2*^ P*

**Supplementary Fig. S1: Fetal urine abundance of 18 inflammatory proteins displaying a non-significant difference between progression to ESKD and no-ESKD (Cohort 1 in Fig. 1).** Abbreviations: KF, kidney failure before the age of 2 years; no-KF, absence of kidney failure before the age of 2 years.

**Supplementary Fig. S2: Fetal urine abundance of CCL2, CXCL9 and CCL4 after normalization for the total fetal urine protein content of patients with PUV in the discovery cohort (Cohort 1 in Fig. 1).** Abbreviations: ** p<0.01; * p<0.05; Abbreviations: ns, non-significant; KF, kidney failure before the age of 2 years; no-KF, absence of kidney failure before the age of 2 years.

**Supplementary Fig. S3:** Zoom (right panels) on the cut-off value (Youden index defined in the discovery cohort) used to calculate the sensitivity and specificity of the 3 cytokines in the validation cohort.

**Supplementary Fig. S4:** Fetal urinary cytokines were not associated to ultrasound abnormalities including amniotic fluid volume (AF) and kidney abnormalities including hyperechogenicity cysts or hypoplasia, except a limited association (p=0.03) of CCL2 abundance with the absence of amniotic fluid.

**Supplementary Fig. S5: Abundance of selected chemokines in amniotic fluid of CAKUT patients (Cohort 4 in Fig. 1).** The abundance in amniotic fluid of CCL2, CXCL9 and CCL4 and the fetal urine negative control inflammatory protein CXCL10 was not different in fetuses with early KF versus fetuses not developing postnatal KF. Abbreviations: ns, non-significant; KF, kidney failure before the age of 2 years; no-KF, absence of kidney failure before the age of 2 years.

**Supplementary Table S1**

Pairwise comparison of ROC curves of CCL2, CXCL9, CCL4, the combination of the CCL2/CXCL9/CCL4 and the 12PUV signature in the validation cohort (Cohort 2 in **Fig. 1**). **a)** None of the comparisons of the different ROC curves display a significantly different AUC. **b)** AUCs and confidence intervals. All analyses were performed with Medcalc (Version 20, 64 bit).

**Supplementary Table 1a) Pairwise comparison of ROC curves**

| CCL2 ~ CXCL9 | |
| --- | --- |
| Difference between areas | 0.0428 |
| Standard Error ^a^ | 0.0774 |
| 95% Confidence Interval | -0.109 to 0.195 |
| z statistic | 0.552 |
| Significance level | P = 0.5808 |
| CCL2 ~ CCL4 | |
| Difference between areas | 0.0987 |
| Standard Error ^a^ | 0.0736 |
| 95% Confidence Interval | -0.0457 to 0.243 |
| z statistic | 1.340 |
| Significance level | P = 0.1803 |
| CCL2 ~ Combo_CCL2_CXCL9_CCL4 | |
| Difference between areas | 0.118 |
| Standard Error ^a^ | 0.0889 |
| 95% Confidence Interval | -0.0557 to 0.293 |
| z statistic | 1.333 |
| Significance level | P = 0.1826 |
| CCL2 ~ 12PUV | |
| Difference between areas | 0.217 |
| Standard Error ^a^ | 0.138 |
| 95% Confidence Interval | -0.0536 to 0.488 |
| z statistic | 1.572 |
| Significance level | P = 0.1160 |
| CXCL9 ~ CCL4 | |
| Difference between areas | 0.0559 |
| Standard Error ^a^ | 0.0624 |
| 95% Confidence Interval | -0.0664 to 0.178 |
| z statistic | 0.896 |
| Significance level | P = 0.3703 |
| CXCL9 ~ Combo_CCL2_CXCL9_CCL4 | |
| Difference between areas | 0.0757 |
| Standard Error ^a^ | 0.0514 |
| 95% Confidence Interval | -0.0250 to 0.176 |
| z statistic | 1.473 |
| Significance level | P = 0.1408 |
| CXCL9 ~ 12PUV | |
| Difference between areas | 0.174 |
| Standard Error ^a^ | 0.0946 |
| 95% Confidence Interval | -0.0111 to 0.360 |
| z statistic | 1.843 |
| Significance level | P = 0.0653 |
| CCL4 ~ Combo_CCL2_CXCL9_CCL4 | |
| Difference between areas | 0.0197 |
| Standard Error ^a^ | 0.0410 |
| 95% Confidence Interval | -0.0605 to 0.100 |
| z statistic | 0.482 |
| Significance level | P = 0.6298 |
| CCL4 ~ 12PUV | |
| Difference between areas | 0.118 |
| Standard Error ^a^ | 0.120 |
| 95% Confidence Interval | -0.117 to 0.354 |
| z statistic | 0.985 |
| Significance level | P = 0.3247 |
| Combo_CCL2_CXCL9_CCL4 ~ 12PUV | |
| Difference between areas | 0.0987 |
| Standard Error ^a^ | 0.0917 |
| 95% Confidence Interval | -0.0810 to 0.278 |
| z statistic | 1.076 |
| Significance level | P = 0.2818 |

**Supplementary Table S1b AUCs and confidence intervals**

| Variable | AUC | SE ^a^ | 95% CI ^b^ |
| --- | --- | --- | --- |
| CCL2 | 0.750 | 0.127 | 0.547 to 0.895 |
| CXCL9 | 0.793 | 0.0915 | 0.594 to 0.923 |
| CCL4 | 0.849 | 0.112 | 0.659 to 0.956 |
| Combo_CCL2_CXCL9_CCL4 | 0.868 | 0.0841 | 0.683 to 0.967 |
| 12PUV | 0.967 | 0.0290 | 0.817 to 0.999 |

^a^ DeLong et al., 1988

^b^ Binomial exact
