# Supplementary material for: Evaluation of predictive performance of fetal urinary inflammatory markers of postnatal kidney function in fetuses with posterior urethral valves
Source: Pediatr Nephrol. 2024 Nov 30;40(4):1023–32. doi: 10.1007/s00467-024-06608-x (PMC11885327; doi:10.1007/s00467-024-06608-x)
Supplement: Supplementary file 2 — Graphical abstract (PPTX 146 KB) [file 467_2024_6608_MOESM2_ESM.pptx]

## Slide 1
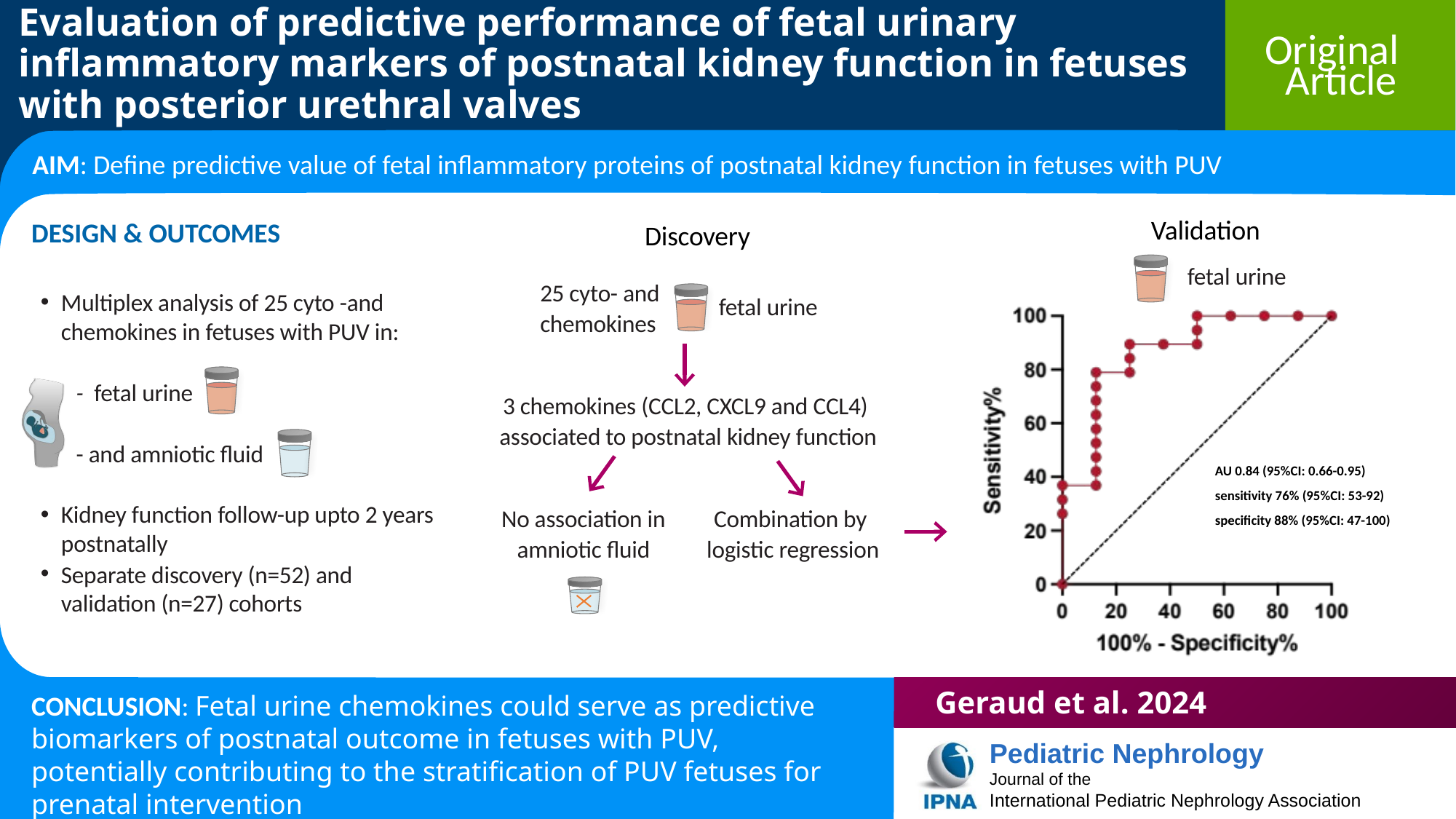

Evaluation of predictive performance of fetal urinary inflammatory markers of postnatal kidney function in fetuses with posterior urethral valves
AIM: Define predictive value of fetal inflammatory proteins of postnatal kidney function in fetuses with PUV
DESIGN & OUTCOMES
Validation
Discovery
fetal urine
25 cyto- and
chemokines
Multiplex analysis of 25 cyto -and chemokines in fetuses with PUV in:
- fetal urine
- and amniotic fluid
Kidney function follow-up upto 2 years postnatally
Separate discovery (n=52) and validation (n=27) cohorts
fetal urine
3 chemokines (CCL2, CXCL9 and CCL4)
associated to postnatal kidney function
AU 0.84 (95%CI: 0.66-0.95)
sensitivity 76% (95%CI: 53-92)
specificity 88% (95%CI: 47-100)
No association in
amniotic fluid
Combination by
logistic regression
 Geraud et al. 2024
CONCLUSION: Fetal urine chemokines could serve as predictive biomarkers of postnatal outcome in fetuses with PUV, potentially contributing to the stratification of PUV fetuses for prenatal intervention
-----
